# E-cigarettes: Effects in phagocytosis and cytokines response against *Mycobacterium tuberculosis*

**DOI:** 10.1371/journal.pone.0228919

**Published:** 2020-02-10

**Authors:** Andromeda-Celeste Gómez, Pablo Rodríguez-Fernández, Raquel Villar-Hernández, Isidre Gibert, Beatriz Muriel-Moreno, Alicia Lacoma, Cristina Prat-Aymerich, Jose Domínguez

**Affiliations:** 1 Servei de Microbiologia, Hospital Universitari Germans Trias i Pujol, Institut d’Investigació en Ciències de la Salut Germans Trias i Pujol, CIBER Enfermedades Respiratorias (CIBERES), Badalona, Catalonia, Spain; 2 Institut de Biotecnologia i de Biomedicina (IBB), Universitat Autònoma de Barcelona (UAB), Bellaterra (Cerdanyola del Vallès), Barcelona, Catalonia, Spain; 3 Departament de Genètica i de Microbiologia, Universitat Autònoma de Barcelona (UAB), Cerdanyola del Vallès, Barcelona, Catalonia, Spain; The University of Georgia, UNITED STATES

## Abstract

Cigarette smoking and tuberculosis are a significant cause of death worldwide. Several epidemiological studies have demonstrated cigarette smoking is a risk factor for tuberculosis. Electronic cigarettes have recently appeared as a healthier alternative to conventional smoking, although their impact in tuberculosis is not well understood. The aim of this study was to explore the effect of electronic cigarettes in phagocytosis of *Mycobacterium tuberculosis* and cytokines production. *In vitro* infection was carried out by exposing THP-1 macrophages to four electronic vapor extracts and the intracellular burden of *M*. *tuberculosis* was determined. The percentage of infection was evaluated by confocal microscopy and the cytokine production by Luminex. A reduction of intracellular *M*. *tuberculosis* burden in THP-1 macrophages was found after its exposure to electronic vapor extract; the same trend was observed by confocal microscopy when *Mycobacterium bovis* BCG-GFP strain was used. Electronic cigarettes stimulate a pro-inflammatory cytokine response. We conclude that electronic cigarettes impair the phagocytic function and the cytokine response to *M*. *tuberculosis*.

## Introduction

Both cigarette smoking and tuberculosis (TB) are a significant cause of death worldwide. The World Health Organization (WHO) estimates that 1.3 and 7 million people died in 2017 of TB and cigarette smoking respectively [1, 2]. It is noteworthy that countries with a high incidence of TB tend to also have an elevated proportion of smokers [3]. The link between TB and cigarette smoke has already been suggested since 1918 and it has been confirmed by several epidemiological studies [4, 5]. There is evidence that cigarette smoke is a risk factor for TB, aiding the infection, the progression to the active disease and the severity of the illness [6, 7]. In addition, it has been observed that cigarette smoke delays in the culture conversion during TB treatment, increasing the risk of transmission to the population [8, 9].

Many studies have been focused on understanding the cause-effect relationship between cigarette smoke and TB, however, many aspects currently remain unclear. Cigarette smoke effects include: loss of phagocytic capacity, failure to contain the intracellular growth of mycobacteria in macrophages, attenuation of cytokine and chemokine response as well as attenuation of apoptosis [10–13].

In the last years, Electronic Nicotine Delivery Systems (ENDS) and especially electronic cigarettes (e-cigs) have appeared as a cooler, cheaper and healthier alternative to conventional tobacco, proving more attractive to the younger population. However, in the medical community there is a growing concern due to: i) the health effects from ENDS, ii) the inexact labelling of the electronic cigarette liquid (e-liquid) components, iii) the lack of international laws for quality production, iv) non-smokers being converted by e-cigs into dual users [14–16].

E-cigs deliver an aerosol (known as vapor) from e-liquids that can contain propylene glycol (PG), vegetable glycerol (VG), flavors and nicotine in different concentrations and ratios [17]. Although the percentage of toxic compounds found in e-cig vapor (e-vapor) are lower than cigarette smoke [17], some studies have shown a toxic effect on cells like alveolar macrophages [18], lung epithelial cells [19–22] and monocytic cells [23] as well as proteomic changes in the cells of the lower airways [24] and pro-inflammatory response [19, 21, 23, 25]. Moreover, function damage in macrophages has been observed and therefore an enhancement of infections caused by microorganisms such as *Haemophilus influenzae* [26], *Escherichia coli* [18], *Staphylococcus aureus* [18, 21] and *Streptococcus pneumoniae* [27]. In addition, it has been shown that e-cigs promote influenza A virus infection in mice [27].

To obtain a better understanding of the relation between TB and e-vapor, we studied the influence of them on phagocytosis of *Mycobacterium tuberculosis* and cytokines production of infected THP-1 macrophages.

## Materials and methods

### THP-1 cell line and mycobacteria growth conditions

The human monocytic cell line THP-1 (ATCC TIB-202^™^) was maintained in RPMI 1640 GlutaMax (Gibco, Paisley, UK) supplemented with 10% heat-inactivated fetal bovine serum at 37°C in 5% CO_2_. The cells were passaged every 3 days. THP-1 monocytes were stimulated to macrophages using 0.1 μM of Phorbol 12-Myristate 13-Acetate (PMA, Sigma, St Louis, USA) for 72 hours (37°C in 5% CO_2_). RPMI was replaced by fresh medium 24 hours before the experiments.

*M*. *tuberculosis* H37Rv and *Mycobacterium bovis* BCG expressing green fluorescent protein (BCG-GFP) strains were grown in Middlebrook 7H9 supplemented with 0.05% tween 80 and 10% albumin-dextrose-catalase (ADC), or on 7H10 agar supplemented with 10% oleic acid-albumin-dextrose-catalase (OADC). For *M*. *bovis* BCG-GFP, 0.08% of glycerol and 20 μg/mL of kanamycin were also added. The mycobacteria cultures were incubated at 37°C. *M*. *bovis* BCG-GFP was kindly provided by Carlos Martín from the University of Zaragoza, Spain.

### Cigarette smoke and e-vapor extraction

Cigarette smoke extract (CS extract) was prepared from commercial cigarettes (Marlboro: 10 mg Tar, 0.8 mg Nicotine, Philip Morris Sàrl Neuchâtel, Switzerland) as was previously reported [28]. Briefly, one cigarette was combusted using a syringe-modified apparatus, which draws the smoke into a sterile glass containing 10 mL of RPMI medium or 7H9-tween. 60 mL of smoke was extracted for 10 sec following a 30 sec break; this process was repeated six times per cigarette and the CS extract was sterilized using a 0.22 μm filter. The resulting solution was considered as a 100% CS extract. The absorbance (320nm) was measured for each batch to assure reproducibility and adjusted to 1.2 ± 0.2 [29, 30]. The working solution was 10% CS extract, equivalent to the smoke of between half a pack and 2 packs of cigarettes per day [31]. For each experiment, fresh extract was used and added to the cultures within 30 min of preparation.

For e-vapor extract, a similar protocol was carried out. QHIT e-cigarette (Puff, Moncalieri, Italy) was used with a Cartomicer CE4 device, 3.7 V. The e-liquid base was a compound of 55% PG: 45% VG. The e-liquid base was used with nicotine (8 mg/g) and without, or with Coffee flavour (Irish Cloud) with and without nicotine (8 mg/g) (Puff It, Puff, Moncalieri, Italy). Coffee flavor was chosen because of the toxic effect observed in fibroblasts [32]. 60 mL of e-vapor was extracted for 3 sec, after which a 30 sec break followed; the process was repeated 40 times (average of puffs found in literature), in 10 mL of RPMI medium or 7H9-tween [21, 26]. The e-vapor extract was also sterilized using a 0.22 μm filter. Like for CS extract, fresh extract was used and added to cultures within 30 min of preparation at 100% concentration according to literature [21, 26].

### Cytotoxicity assay

To determinate the cytotoxic effect of the cigarette smoke and e-vapor extracts on THP-1 macrophages, EZ4U (Biomedica, Vienna, Austria) assay was used following the manufacturer’s instructions. THP-1 monocytes were seeded at a concentration of 1 × 10^5^ cells per well in 96-well tissue culture plates with clear bottoms (Falcon^®^, Tewksbury, USA) using RPMI without phenol red containing PMA. The plates were incubated for 72 h (37°C in 5% CO_2_ atmosphere). CS extract or e-vapor extract were diluted in 200 μL of RPMI without phenol red per well at different concentrations and incubated for 3 hours for CS extract and 24 hours for e-vapor extract. The monolayers were washed once with Dulbecco’s PBS (DPBS) and then 200 μL of fresh medium without phenol red and 20 μL of EZ4U were added. The absorbance was read after 4 h of incubation (day 0), and also at days 3 and 6. For the plates of day 6, medium was changed on day 3. A microplate reader (Victor 3, Wallac, Waltham, USA) was used with a wavelength of 450 nm with 620 nm as reference. The results are expressed as viability percentage using cells unexposed as a control.

### Macrophage infection with *M*. *tuberculosis*

THP-1 macrophages were exposed to CS or e-vapor extract, and then infected with *M*. *tuberculosis* following a protocol previously reported [33], with some modifications. Briefly, 3 × 10^5^ cells per well were PMA stimulated and seeded in 24-well tissue culture plates with clear bottoms (Falcon^®^). Freshly prepared 10% CS extract or 100% e-vapor extract were added to the cells and incubated for 3 hours for CS extract and 24 hours for e-vapor extract at 37°C in 5% CO_2_. For the infection, mid-log phase *M*. *tuberculosis* were washed twice with DPBS+ 0.05% tween and subsequently once with DPBS after which they stood for 5 min, before the supernatant were collected. The bacteria were then diluted in RPMI with CS extract or e-vapor extract and added to the THP-1 macrophages at a multiplicity of infection (MOI) of 0.1. After 3 h of contact at 37°C in 5% CO_2_, the macrophages were treated with 200 μg/mL amikacin for 1 h and washed three times with DPBS to eliminate any extracellular bacteria. Lastly, 1 mL of RPMI with CS extract or e-vapor extract was added to each well and incubated at 37°C in 5% CO_2_. Fresh medium with CS extract or e-vapor extract was added at day 3. Intracellular growth was assessed by lysis of the monolayers by the addition of 500 μL of water followed by a 30 min incubation at room temperature and serial dilution in PBS-tween plating onto Middlebrook 7H10 solid medium at days 0, 1, 2, 3 and 6. Colonies were counted after 3–4 weeks incubation at 37°C and the average CFUs/mL determined.

### Confocal microscopy

THP-1 macrophages were seeded on a 12mm circular coverslips in 24-well plate. The infection was performed as described above but using *M*. *bovis* BCG-GFP strain or 1-μm-diameter yellow green latex beads (Sigma, St Louis, USA) with a MOI of 10. After infection, cells were fixed overnight with 4% paraformaldehyde (PFA). Coverslips were washed twice with DPBS and incubated with DPBS-BSA 1% 10 min. For staining, coverslips were washed once with DPBS and then 200 μL of DPBS containing Hoechst 33342 (1:1000) and texas red-X-phalloidin (1:200) were added and incubated for 10 min. Hoechst and red-X-phalloidin were purchased in Invitrogen (Waltham, USA). Finally, the coverslips were washed once with DPBS, once with water and subsequently mounted with Prolong Gold Antifade Reagent (Invitrogen). For quantification, 900 cells from three independent experiments (300 cells/coverslip) were counted per day and treatment. The images were taken with an Olympus Fluoview 1000 microscope at days 0, 3 and 6 for the experiments with *M*. *bovis* BCG-GFP and at day 0 for the latex beads experiments. The images were analysed using ImageJ software [34]. Latex beads were used in order to determine if phagocytosis was impaired in a general or specific mycobacteria pathway.

### Cytokines detection

Cytokines production from infected and uninfected THP-1 macrophages, which were either exposed or non-exposed to e-vapor extract or CS extract, were assessed using the human magnetic luminex assay kit (LXSAHM, R&D Systems, Minneapolis, USA) according to the manufacturer’s instructions. Analysis included detection of some of the most important cytokines involved in the immune response against *M*. *tuberculosis* infection: tumour necrosis factor (TNF)-α, interleukin (IL)-6, IL-8, IL-10, IL-12, IL-18, IL-1β and interferon (IFN)-γ. Supernatants were recovered from infection assays with *M*. *tuberculosis* H37Rv strain and sterilized using a 0.22 μm filter. For the supernatants of day 6, 500 μL extra of fresh RPMI (with or without e-vapor extract or CS extract) were added on day 3 (this extra volume was taken in account in the calculation of cytokine production). Cytokine levels were calculated using the Luminex^®^ 200^™^ system and the xPONENT^®^ 3.1 software (Luminex Technologies, Inc., Austin, USA).

### Statistical analyses

Statistical analyses were performed using the GraphPad PRISM 6.0 software package (San Diego, California, USA). Differences between treatments were compared by the Mann-Witney test. A *p*-value ≤0,05 was considered as a statistically significant.

## Results

### E-vapor extract and cigarette smoke extract are not toxic for THP-1 and mycobacteria at the concentrations used

**Fig 1:**
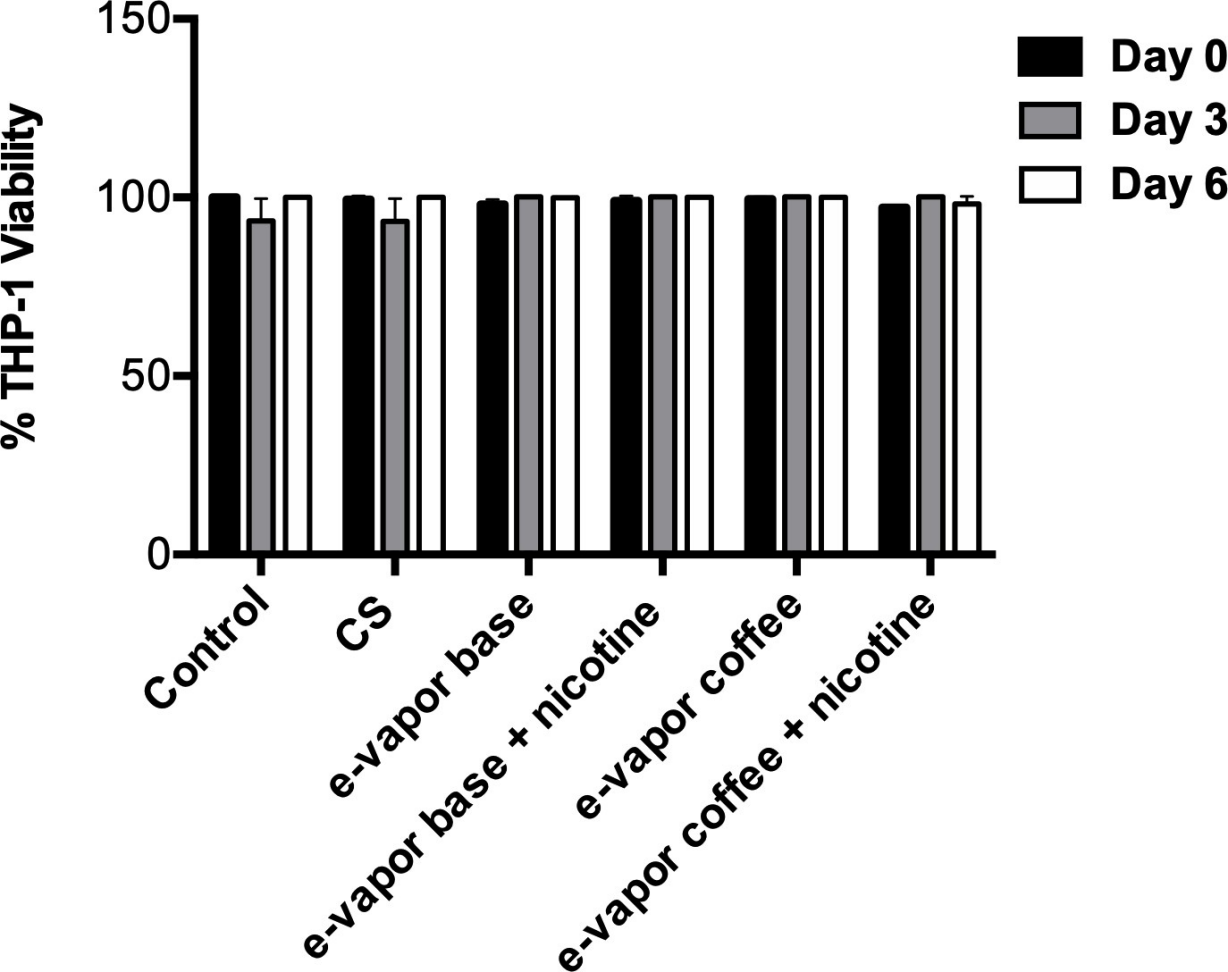
THP-1 viability after exposition to cigarette smoke (CS) extract and e-vapor extract. Formazan-based test EZ4U was performed at 4 hours (day 0), 3 and 6 days of exposition to 10% CS extract, 100% e-vapor base extract with and without nicotine and e-vapor coffee flavor extract with and without nicotine. The viability percentage was calculated using cells unexposed as a control. The results are expressed as the average and standard deviation of six replicates of at least two independent experiments.

We evaluated if the concentrations found in literature: 10% CS extract and 100% e-vapor extract were nontoxic in THP-1 macrophages [21, 26]. The macrophages were exposed to e-vapor or CS extracts for 6 days and the cell viability was assessed using the formazan-based cell proliferation assay EZ4U at days 0, 3 and 6. The assessed concentrations assessed of CS extract and e-vapor extract (both non-flavored and coffee flavored on a base with or without nicotine) did not affect the viability of THP-1 (Fig 1).

**Fig 2:**
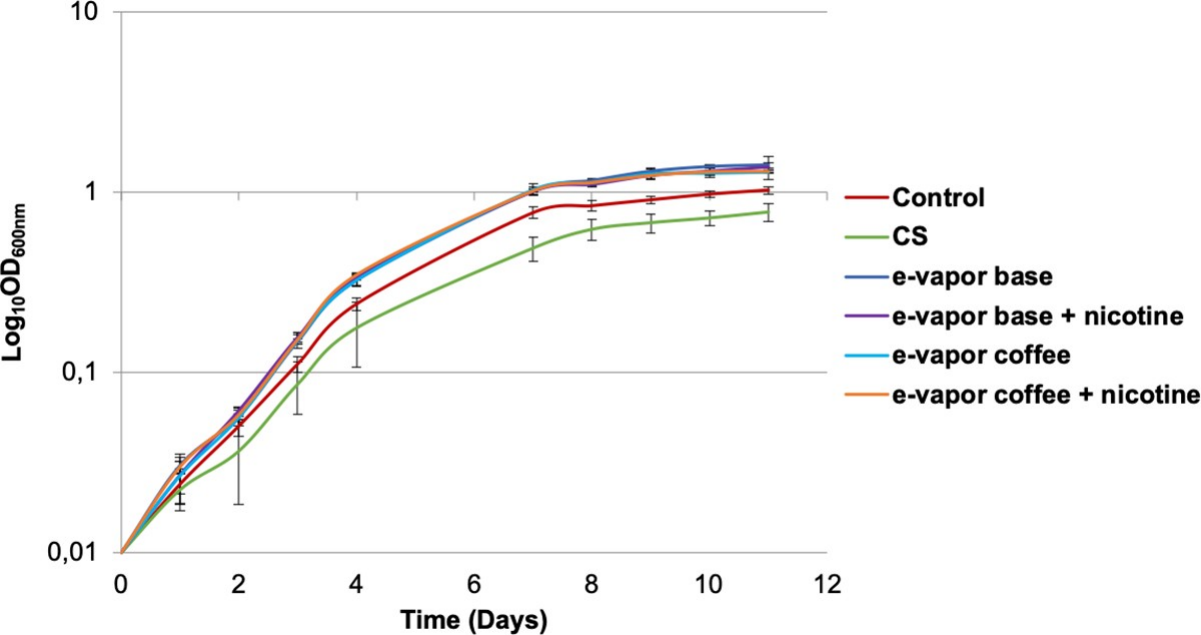
Growth of *M*. *tuberculosis* H37Rv exposed to 10% CS extract, 100% e-vapor base extract with and without nicotine and e-vapor coffee flavor extract with and without nicotine. The results are expressed as the average and standard deviation of duplicates of at least two independent experiments.

On the other hand, we studied the effect of e-vapor extract and CS extract on *M*. *tuberculosis* H37Rv growth. The cultures were exposed to e-vapor extract and CS extract for two weeks and the OD_600nm_ was measured every 24 hours. The results obtained showed no significant changes. A slight increase in the growth of *M*. *tuberculosis* was observed when it was exposed to e-vapor extract and a slight decrease when the bacteria were exposed to CS extract (Fig 2).

### E-vapor and cigarette smoke extracts reduce the phagocytosis of *M*. *tuberculosis* by THP-1 macrophages

**Fig 3:**
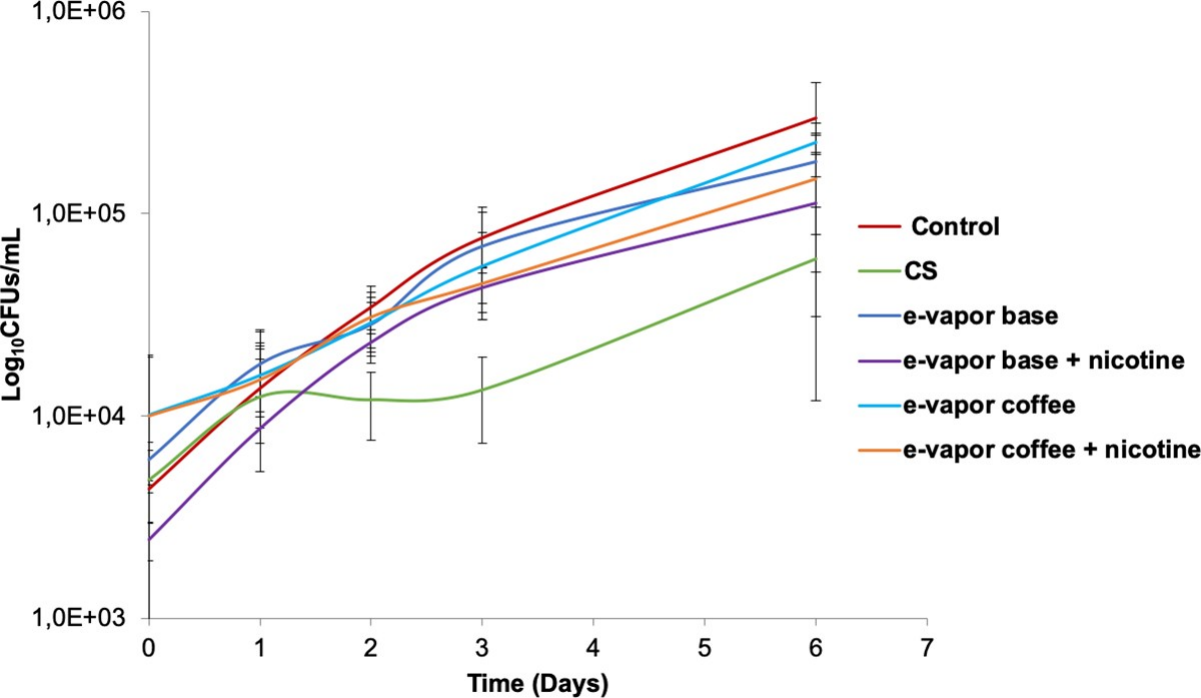
*M*. *tuberculosis* intracellular CFUs counts over 6 days. THP-1 macrophages exposed to 10% CS extract, 100% e-vapor base extract with and without nicotine and e-vapor coffee flavor extract with and without nicotine. The results are expressed as the average and standard deviation of triplicates of at least three independent experiments.

As was previously reported in literature for other microorganisms, we studied if the extracts of e-vapor and cigarette smoke had any effect in the phagocytic function after infection with *M*. *tuberculosis*. Therefore, macrophages were exposed to e-vapor extract and CS extract for 24 hours and 3 hours respectively, and then infected with H37Rv strain. The intracellular CFUs were evaluated during 6 days. When the macrophages were exposed to e-vapor extract base and e-vapor extract base with nicotine, a reduction of the number of viable intracellular *M*. *tuberculosis* was observed compared with the control. Less mycobacteria were recovered when the cells were exposed to e-vapor extract base with nicotine compared with e-vapor extract base without nicotine. A similar tendency was observed when the macrophages were exposed to e-vapor extract with coffee flavor, however the CFUs counts were higher than e-vapor extract base. The highest decrease in the CFUs counts were observed when the macrophages were exposed to CS extract compared with e-vapor extract and control. The highest decrease in the CFUs counts were observed when the macrophages were exposed to CS extract compared with e-vapor extract and control (Fig 3).

**Fig 4:**
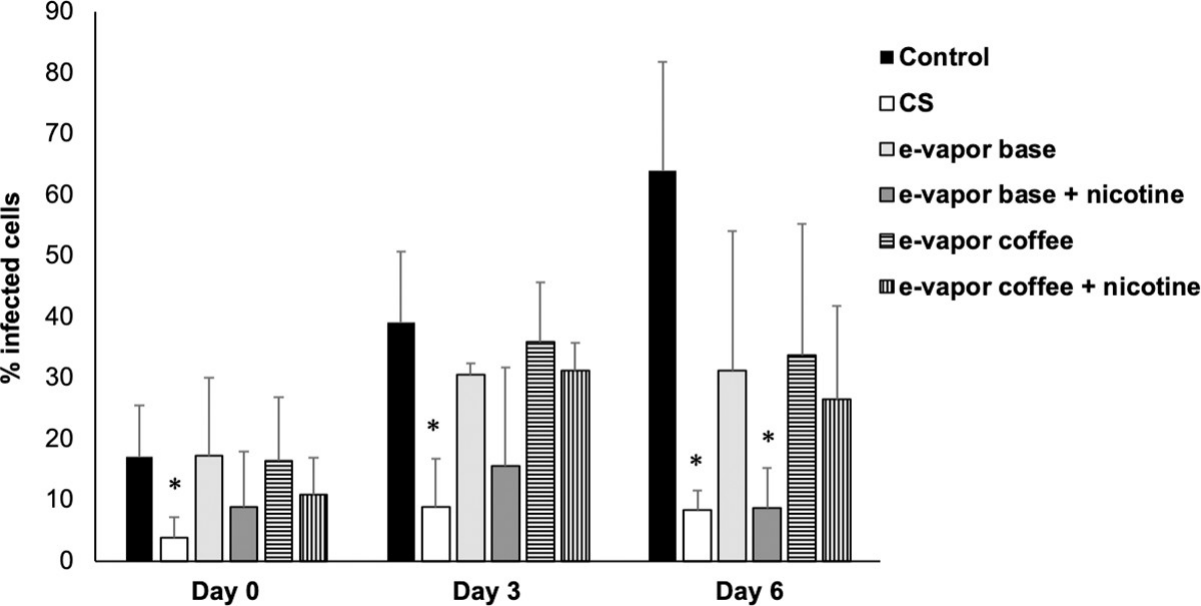
Phagocytosis of *M*. *bovis* BCG-GFP by THP-1 macrophages. Macrophages were exposed 10% CS extract, 100% e-vapor base extract with and without nicotine and e-vapor coffee flavor extract with and without nicotine. The results are expressed as the average and standard deviation of triplicates of at least three independent experiments using confocal microscopy. Significance from control, *P*<0,05, Mann-Whitney *U* test.

In order to confirm the results obtained in CFUs counts, we determined the percentage of infected cells using confocal microscopy. The fluorescent strain *M*. *bovis* BCG-GFP was used, following the same infection protocol used with *M*. *tuberculosis*. As was observed in the CFUs counts, the results show that there is a significant reduction in the uptake of *M*. *bovis* BCG-GFP when the macrophages are exposed to CS extract compared with the control (Fig 4). Moreover, there is a trend toward an increasing in the uptake of mycobacteria after the exposition to e-vapor extract compared with CS extract, but to a lesser extent than the control sample (Fig 4). Subsequently, the results obtained by confocal microscopy confirm the results obtained by CFUs counts.

**Fig 5:**
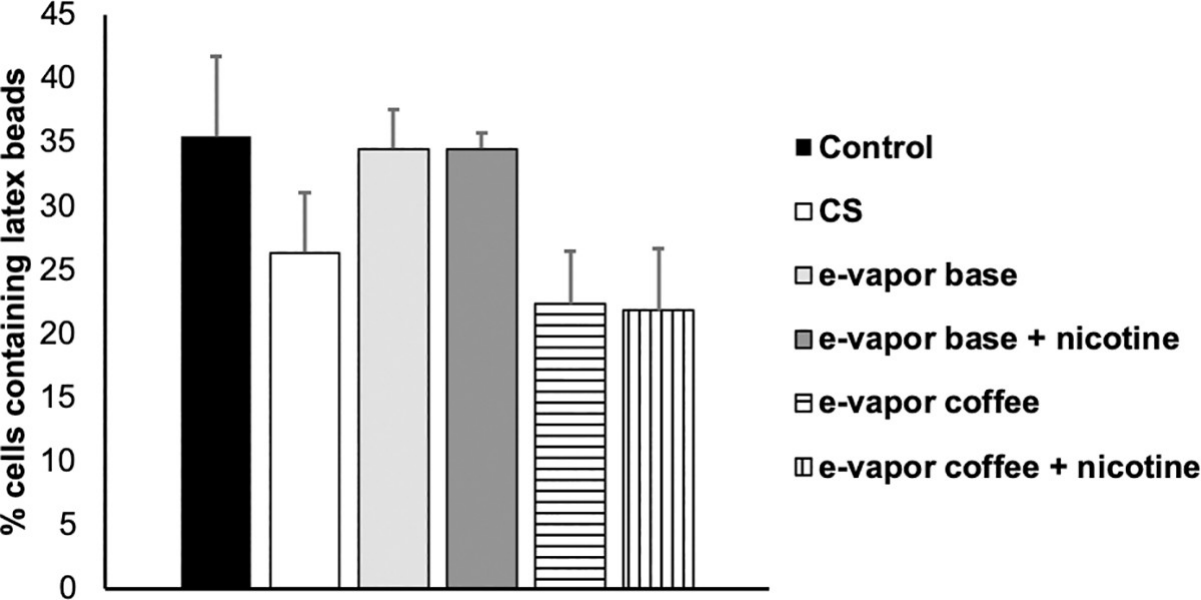
Phagocytosis of GFP-latex beads by THP-1 macrophages. Macrophages were exposed to 10% CS extract, 100% e-vapor base extract with and without nicotine and e-vapor coffee flavor extract with and without nicotine. 300 cells were counted. The results are expressed as the average and standard deviation of at least two independent experiments using confocal microscopy.

To determinate if the reduction of mycobacteria uptake is due to a general or specific defect in the phagocytic function, we evaluated the effect of CS extract and e-vapor extract in the capacity to phagocyte inert material like latex beads. Macrophages were exposed to e-vapor or CS extracts and then the latex beads were in contact during 3 hours. The images were analyzed and the results show no major changes in phagocytosis, suggesting a specific defect in some specific pathway when the cells are exposed to CS extract (Fig 5). Less macrophages with latex beads inside were observed when the cells were exposed to e-vapor coffee extract with and without nicotine (Fig 5).

### E-vapor extract and cigarette smoke extract alter cytokine production

**Fig 6:**
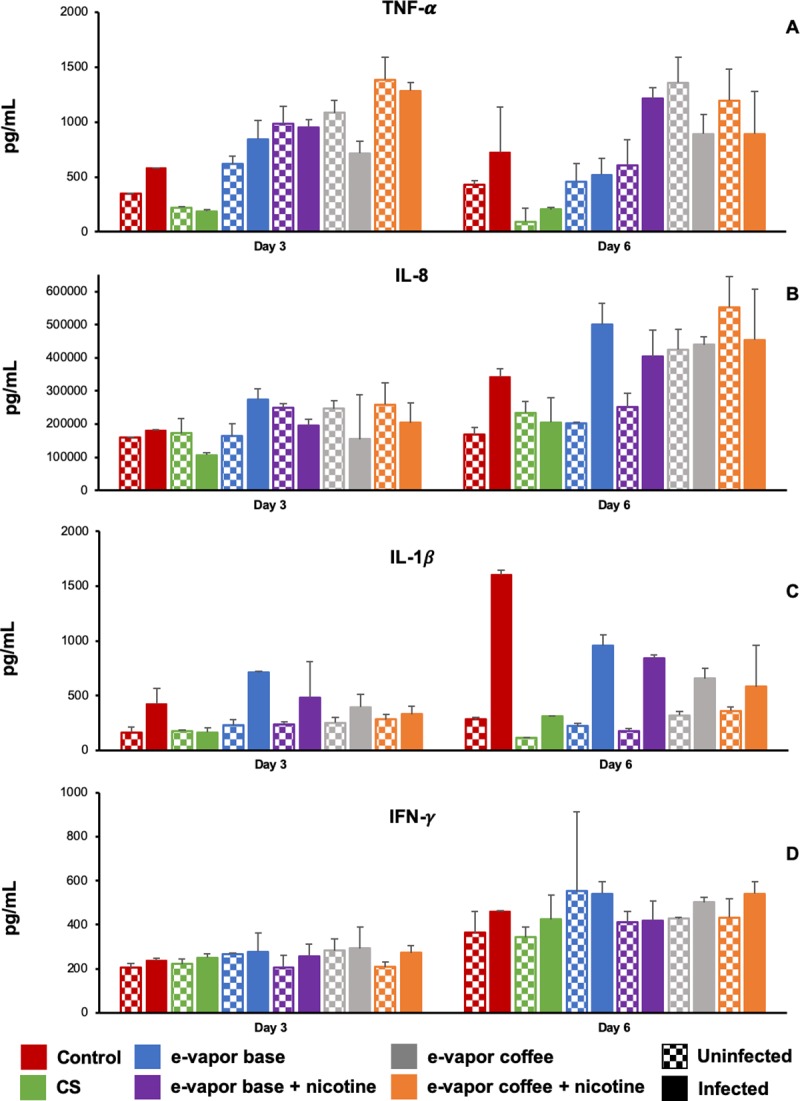
Cytokine production by THP-1 exposed and non-exposed to e-vapor extract and CS extract. Macrophages were exposed to 10% CS extract, 100% e-vapor base extract with and without nicotine and e-vapor coffee flavor extract with and without nicotine. Macrophages were uninfected or infected with *M*. *tuberculosis* H37Rv strain. (A) tumor necrosis factor (TNF-α). (B) Interleukin 8 (IL-8). (C) interleukin 1-beta (IL-1β). (D) interferon gamma (IFN-γ). The results are expressed as the average and standard deviation of at least two independent experiments.

Given that previous studies have shown that e-vapor extract and CS extract alter the cytokine secretion and consequently the clearance of infections, we explored the cytokine response to e-vapor extract and CS extract in THP-1 macrophages. In general, the tendency observed was that the unexposed macrophages produced more cytokines when they were infected. It was also observed that macrophages exposed to CS produced less cytokine response than unexposed cells or cells exposed to e-cigs (Fig 6).

Regarding e-cigs vapor exposed cells, cytokine production was related to the cytokine and the e-cigs vapor extract used. As a general trend, the cytokine response was higher than the cells exposed to CS and even than unexposed cells. The production of TNF-α was higher in infected cells at day 6 exposed to e-vapor with coffee flavour (with and without nicotine) (Fig 6A). IL-8 is was the cytokine produced in higher amount (Fig 6B). The IL-1β was highly produced by cells exposed to *M*. *tuberculosis* infection (Fig 6C). Finally, the production of IFN-γ was similar between uninfected and infected cells (Fig 6D). IL-6, IL-10, IL-12 and IL-18 were all of them below the detection levels.

**Fig 7:**
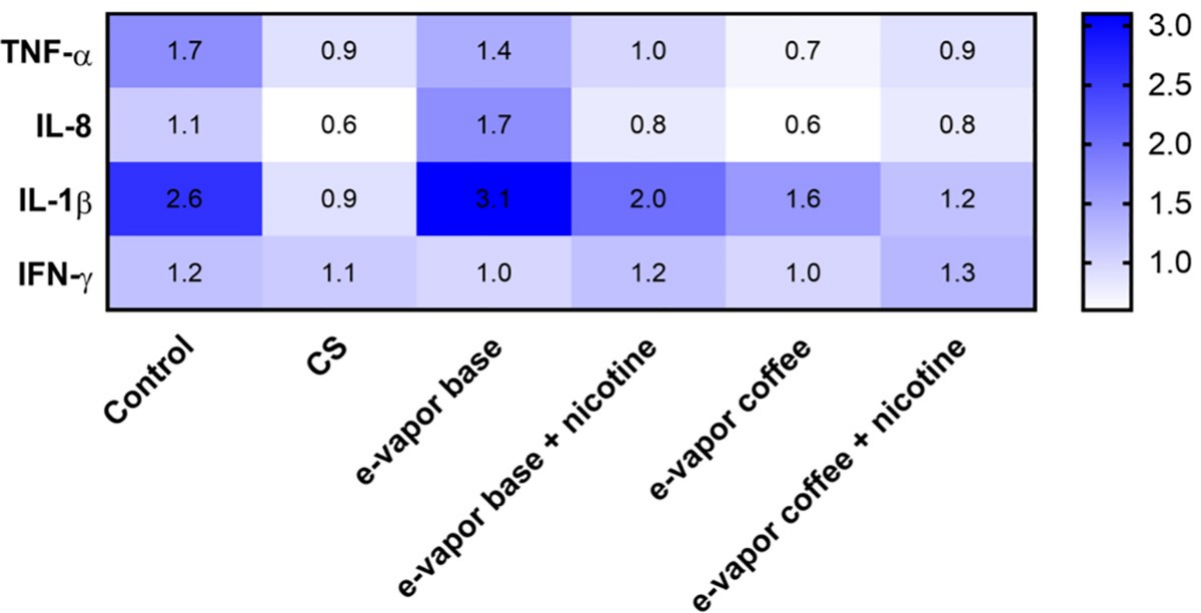
Increment of the cytokine production at day 3 in infected cells compared with the uninfected cells with the same exposure. The numbers in the figure are the result of dividing the amount of cytokine in infected cells between uninfected cells. Values higher than 1.0 mean production of cytokines in infected cells are higher than in uninfected. The higher the number, the higher the increment.

In Fig 7 is shown the ratio between the amount of cytokine produced by each exposed, infected and the non-infected cells. It is observed, that the cells exposed to CS and e- vapor extract with coffee flavor (with and without nicotine) have an impaired response to infection in comparison with unexposed cells and e-cigs base and without flavor or nicotine.

## Discussion

It has been widely proven that CS has an adverse impact in the human health and despite the efforts oriented to reduce its use, it still remains as an issue worldwide. E-cigs have been marketed as a healthier option, however like CS, it has been related with cytotoxicity and abnormal immune response compromising the clearance of pathogens. In this study, we evaluated the impact of e-cigs and CS on the phagocytosis of *M*. *tuberculosis* and cytokines production of infected THP-1 macrophages. Our results show that e-cigs and CS have a deleterious impact in the immune response against TB.

We tested the effect of 100% e-vapor and 10% CS extracts on viability of THP-1 macrophages and we did not observe a cytotoxic effect, this result confirms that e-vapor is less toxic than CS, since it has been reported that higher than 10% concentrations of cigarette smoke are toxic in several cell lines [35, 36]. This was also observed by us when higher concentrations than 10% were tested (data not shown). Free radicals are involved in the tobacco cytotoxicity and they are produced not only in the tobacco combustion, but also when e-liquid is heated in the atomizer [37]. Scott *et al*. detected lower cytotoxicity in alveolar macrophages in the liquid form compared to the vaped [38]. The lower cytotoxicity observed in e-vapor extract can be related with the low concentration of toxicants due to the low temperature of evaporation of the e-liquid compared with the tobacco combustion when a cigarette is smoked [32, 37, 39]. On the other hand, our results are in line with the results obtained by Farsalinos *et al*. and Romagna *et al*. in which the presence of nicotine in e-vapor extract did not affect the cell viability [32, 39]. The results found in literature, regarding the cytotoxicity of e-vapor extract flavored are contradictory; some studies have shown a cytotoxic effect when tobacco, cherry, cinnamon and coffee flavors were tested [32, 40–42]. However, in agreement with our results, Ween *et al*. observed that cytotoxicity in THP-1 macrophages was not related with the e-cig flavor [26]. It has been suggested that cell line used, nicotine, flavors, PG/PV ratio, voltage and potency of the device can influence in the toxicity effects of e-vapor [43].

Innate immune response is crucial for the outcome of TB infection as well as for the adaptive immune response [44]. Macrophages are a pivotal piece of the innate immunity for TB and its response is affected by cigarette smoke and e-vapor. Our results showed a decrease of the intracellular *M*. *tuberculosis* in THP-1 macrophages under the exposition to e-vapor extract as well as CS extract, where even less viable *M*. *tuberculosis* where found compared with the control. Similar trend was obtained by confocal microscopy using BCG-GFP strain. These results suggest that e-vapor extract and CS extract cause a phagocytosis impairment in THP-1. This kind of damage has been observed in monocytes from smokers TB patients [12], in alveolar macrophages from smokers [13], in lung-tissue and alveolar macrophages from smoking mice [45, 46] and in alveolar macrophages and THP-1 macrophages when cells exposed to CS extract were infected with *H*. *influenzae* [26, 36]. The reduction in the phagocytic ability has been related with the decrease of the expression of recognition molecules for apoptotic cells [13], the inhibition proteins involved in cytoskeletal rearrangements [46], the blockage of PI3K signaling cascade [36] as well as the decrease of the expression of bacterial recognition receptors [26, 47, 48]. However, it has been also observed opposite results. Shang *et al*. found an increase of viable intracellular *M*. *tuberculosis* in THP-1 macrophages after the exposition to CS extract (from 3R4F standard cigarette) compared with the macrophages non-treated. In the study of Shang *et al*., an increase in the intracellular burden was also found in alveolar macrophages obtained from autopsies of smokers [10]. Other studies showed similar results using the BCG and H37Ra strains in alveolar macrophages and monocyte-derived macrophages (MDM) [11, 35]. Moreover, Bai *et al*. observed that the nicotine component of CS extract reduces the number of autophagosomes leading an increase of *M*. *tuberculosis* burden in alveolar macrophages [3]. Although, it is important to highlight that the cell line, the strain, the type of cigarette/e-cig, the extraction method, the exposure dose and time all have an influence in the results obtained [49].

On the other hand, in line with our results with cigarette smoke, other studies have shown similar results in e-cigs. In mice model, it has been observed that the exposure to e-vapor extract decreased also the phagocytic ability [27]. Phagocytosis of *H*. *influenzae* is also decreased in THP-1 macrophages by e-vapor flavored with or without nicotine but not with e-vapor base extract, suggesting that flavors could have a role in the phagocytosis impairment [26]. However, our results showed the opposite, as we did not observe that coffee flavor affect more the growth of H37Rv or BCG-GFP inside macrophages exposed to e-vapor extract flavored although in our case, the flavor tested is different from Ween *et al*. [26]. Additionally, our results showed that the macrophages exposed to e-vapor extract with nicotine had less intracellular mycobacteria than the cells exposed to e-vapor extract without nicotine, suggesting that nicotine in e-vapor extract has an effect in the phagocytosis of mycobacteria. On the other hand, Ween *et al*. observed that the impairment of phagocytosis by e-cigs was also linked with the expression of bacterial recognition receptors [26].

It has been observed that CS extract produce damage in specific phagocytosis pathways in alveolar macrophages [36], and in our experience, the results obtained with *M*. *bovis* and with latex beads also suggest the same specific impairment to phagocyte alive particles with both, e-vapor with and without nicotine.

As important mediators of the immune system, macrophages release cytokines in order to regulate both innate and adaptive immunity [50]. Cytokines are produced by macrophages activated by *M*. *tuberculosis* infection [51]. This is in agreement with our results where the release of cytokines is stimulated for the infection in unexposed cells, compared with the uninfected control. It has been observed that another effect of e-cigs and CS on macrophages is the alteration of cytokines production, stimulating both inflammatory and anti-inflammatory responses [45]. TNF-α, IL-1β and IFN-γ are pro-inflammatory cytokines that are important in the host defence after *M*. *tuberculosis* infection [51]. TNF-α also initiates the activation of the phagocyte [51]. Similar to our results, several studies have found that CS extract decrease the production of TNF-α in uninfected alveolar macrophages, THP-1, Ana-1 macrophages, monocyte-derived macrophages and human peripheral blood mononuclear cells (PBMC) [11, 26, 50, 52, 53] and IL-1β also in uninfected THP-1, monocyte-derived macrophages and PBMC [26, 52, 54]. We observed the same tendency in infected cells exposed to CS extract, which produced less TNF-α and IL-1β compared with the infected cells non-exposed to CS extract. Despite some studies have shown that IFN-γ is decreased by CS [8, 35, 55], we did not observe important changes in its release, compared to control in THP-1 macrophages. IL-8 is a neutrophil chemoattractant that mediates the inflammatory process [51] that is increased in uninfected THP-1 macrophages exposed to CS extract [26]. Our results show the same tendency.

The effect of e-vapor extract in the cytokines production is different to the observed in CS extract, with a tendency to produce a pro-inflammatory response. A pattern of elevated cytokine production after e-cig inhalation was observed in mice model [21]. Our results show a trend to increase TNF-α in infected and uninfected cells. Opposite results were obtained by Ween *et al*. where TNF-α was decreased in uninfected THP-1, however the cytokine was measured at 24 hours, when the production could be lower [26] and we speculate that e-vapor extract can require more time in order to see the effect in the cytokines production. Additionally, an increase in the TNF-α production by e-vapor extract in HaCaT cells and by e-cig condensate in THP-1 was observed [18, 22]. On the other hand, the e-vapor extract effect observed on uninfected THP-1 as observed by Ween *et al*. showed a decrease of IL-1β [26] which we did not observe. However, we found this decrease in infected cells mainly on the last day of the experiment. Cervellati *et al*. showed that IFN-γ was increased in uninfected HaCaT cells exposed to e-vapor extract [22], although we observed only a slight increase also the last day of the experiment. Contributing to the inflammation process, IL-8 is increased in infected and uninfected THP-1 macrophages; this finding is similar to several studies in uninfected cells including THP-1 [18, 20, 22, 23, 26].

As it is observed in other studies where the e-cig are used, this study has some limitations: i) the use of only one type of e-cig of second-generation and only one flavour although there is a wide range of e-liquid brands and devices available on the market, ii) as an *in vitro* study, the lung environment is not completely represented, iii) not all experiments were done with virulent *M*. *tuberculosis* strain because of biosafety normatives and cannot be assumed that response of nonvirulent mycobacteria would be the same, iv) it is difficult to estimate if the concentrations and duration of CS and e-vapor are representative of what happens in the smokers [35]. The topography between vaping and cigarette smoking is also different, and e-cigs users could vape more reaching a similar effect to tobacco.

In summary, e-cigs impair the phagocytic function of THP-1 macrophages as well as promote the pro-inflammatory response. Moreover, we confirm that CS affects also the phagocytic function and decreases the effector cytokines response (TNF-α, IL-1β and IL-8). Although e-cigs are advertised as a better option and its effect is not as strong as cigarette smoke, the dependency and long term consequences are not still well known. Our data contributes to explain some of the mechanisms behind the epidemiological link between TB and smoking, and to support the policy efforts to reduce the exposure to smoke and e-vapor as part of the TB control strategies.

## Supporting information

S1 Dataset (XLSX)
